# First person – Andrea Cappa

**DOI:** 10.1242/bio.039644

**Published:** 2018-12-15

## Abstract

First Person is a series of interviews with the first authors of a selection of papers published in Biology Open, helping early-career researchers promote themselves alongside their papers. Andrea Cappa is first author on ‘Live imaging of cortical granule exocytosis reveals that *in vitro* matured mouse oocytes are not fully competent to secrete their content’, published in BiO. At the time of writing the paper, Andrea was a PhD student in the lab of Dr Marcela Michaut at National University of Cuyo, Mendoza, Argentina, investigating biological sciences.


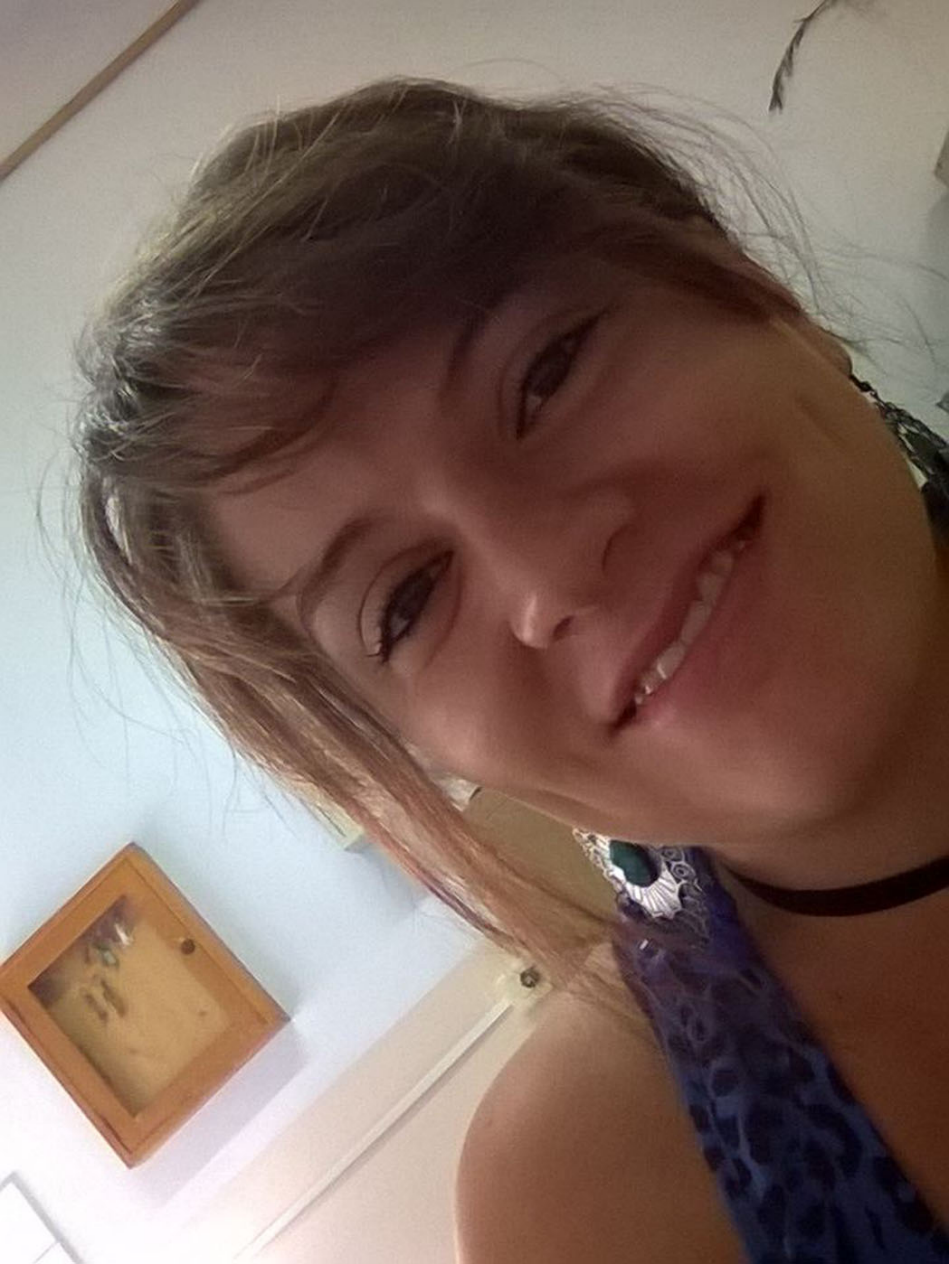


**Andrea Cappa**

**What is your scientific background and the general focus of your lab?**

Our lab is working on analyzing the processes of membrane fusion that occur in gametes during fertilization, as well as in the processes that relate to oocyte maturation and early preimplantation development. My scientific background is in cortical reaction dynamics and their relation to *in vitro* maturation of oocytes.

**How would you explain the main findings of your paper to non-scientific family and friends?**

It is known that *in vitro* maturation cannot mimic the *in vivo* conditions of maturation and this affects oocyte quality. Nevertheless, we don't know what processes are affected. As written in this paper, we saw that one of these processes is the cortical reaction. The cortical reaction is a process initiated during fertilization by the release of cortical granules from the egg, which prevents polyspermy. Most *in vitro* matured oocytes cannot extrude their cortical granules, which may explain their low fertilization rate.

Given the importance of the cortical reaction during fertilization, we think that real time analysis of this process can provide a quick method to evaluate oocyte quality. It can be used, for example, to compare different culture media.

“Given the importance of the cortical reaction during fertilization, we think that real time analysis of this process can provide a quick method to evaluate oocyte quality.”

**Figure:**
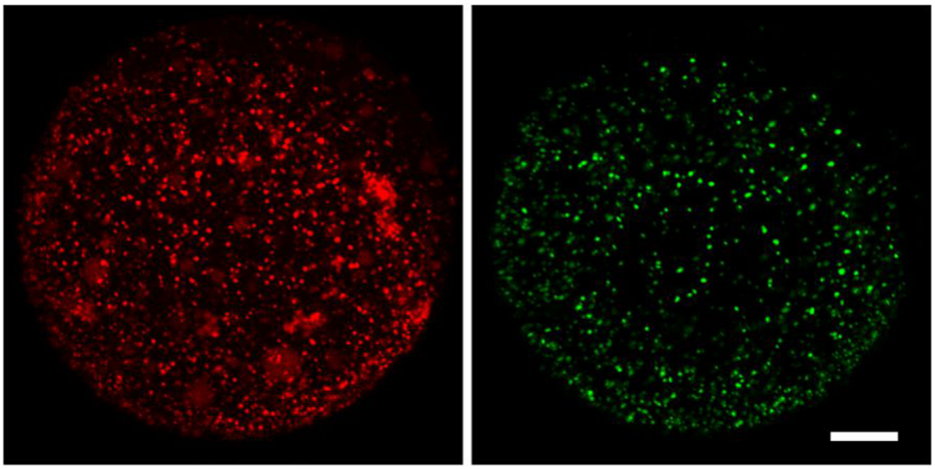
**Cortical reaction.** Reacted ovulated oocyte showing remnant cortical granules (left) and exudate (right). Scale bar: 20µm.

**What has surprised you the most while conducting your research?**

In the beginning, we expected that *in vitro* maturation would affect cortical reaction kinetics. But, surprisingly, we found that most oocytes did not react at all. This indicated to us that the problem was more serious than we initially thought.

**What motivated you to submit your article to BiO?**

BiO is a new, prestigious and accessible journal that has achieved strong impact factor growth.

**What's next for you?**

I hope to continue specializing in animal reproduction.

## References

[BIO039644C1] Cappa A. I., de Paola M., Wetten P., de Blas G. A., and Michaut M. A. (2018). Live imaging of cortical granule exocytosis reveals that *in vitro* matured mouse oocytes are not fully competent to secrete their content. *Biol. Open* 7, bio031872 10.1242/bio.031872 30341105 PMC6310882

